# Age Differences, Age Changes, and Generalizability in Marathon Running by Master Athletes

**DOI:** 10.3389/fpsyg.2019.02161

**Published:** 2019-09-20

**Authors:** Michael John Stones

**Affiliations:** Department of Psychology, Lakehead University, Thunder Bay, ON, Canada

**Keywords:** aging, age trends, human potential, cohort effects, longitudinal trend, physical performance, master athlete

## Abstract

This study examines the world’s Top 100 age class performance times by Master athletes in marathon running. The predominant paradigm for this type of research assumes that the outcomes represent a “virtual” cross-sectional study with important implications about aging. This article critiques this perspective and presents alternative models that include temporal dimensions that relate to cohort differences, age changes and historical transitions. One purpose of this study is to compare these models with respect to goodness of fit to the data. A second purpose is to evaluate the generalizability of findings from the fastest divisional age class quartile to the slower quartiles. Archival listings by the Association of Road Racing Statisticians include a maximum of 100 fastest age class performances in marathon running performances by men and women. This database includes 937 performances by 387 men performances and 856 performances by 301 women. The mean ages are 62.05 years for men and 60.5 years for women. The mean numbers of performances per runner are 6.64 for men and 6.4 for women. Analysis by mixed linear modeling (MLM) indicates best goodness of fit for logarithms of performance time by a model that includes linear and quadratic expressions of age at entry into the database (termed “entry cohort”) and subsequent age changes (termed “elapsed age”) as variables. Findings with this model show higher performance times in women than men. Rates of increase in performance time are higher at older cohort ages and elapsed ages. Performance time increases with interactions between cohort age and elapsed age, cohort age and gender, and elapsed age and gender (i.e., with greater increases in women than men). Finally, increases in performance time with cohort age and elapsed age are higher in slower than faster performance quartiles, with athletes in the faster quartiles more likely to have multiple data entries and athletes in the slower quartiles single data entries. Implications of these findings are discussed.

## Introduction

What Stones and Kozma (1985) and Rittweger et al. (2009) term optimum physical performance encompasses the study of age trends in peak athletic performance. These studies began in the mid-1970s, about a decade after the emergence of competitions for athletes “past their prime” (i.e., aged 40 years and older) – now known as master athletes (Weir et al., 2010). The earliest studies include analyses of running (Moore, 1975; Salthouse, 1976; Stones and Kozma, 1980), track and field athletics (Stones and Kozma, 1981) and swimming (Rahe and Arthur, 1975; Hartley and Hartley, 1984; Stones and Kozma, 1984). The data for the majority of those studies derive from archival listings of age class records, yearly performance rankings, and medal-winning performances in major championships. The main forms of analysis include simple regressions of performance on age, followed by comparison of the age trends across events.

Rittweger et al. (2009) provide a cogent summary of assumptions about the methodology and applied significance of the preceding approach to modeling human performance potential. First, they propose that “world records … can be regarded as results of one of the largest human experiments ever accomplished, taking into account the large underlying sample group, the well-defined criteria and the meticulous surveillance involved” (p. 683). Second, “record data from master athletes can be regarded as a ‘virtual’ cross-sectional study that can provide important insights into the aging process” (p. 683). Third, master athletes “may be regarded as a model for ‘successful’ aging” because “they allow assessment of the relative contribution of senescence (i.e., an irreversible biological process) as opposed to sedentarism or co-morbidity” (p. 683).

This author agrees that peak performances by master athletes provide a unique opportunity to study human physical performance potential with a rigor that satisfies the exacting standards of good science. However, questions can be raised about the validity of the other assumptions. For example: do “virtual” cross-sectional age trends provide an adequate representation of effects due to aging? Does an answer to the preceding depend on the type of database available for analysis? Are the traditional statistical procedures still appropriate for analytic purposes? Do age trends in peak performance generalize to those at lower ability levels? The following paragraphs address these concerns.

### Temporal Trends Relevant to Aging Research

Cross-sectional trend is one of three temporal trends that Schaie (1977) identifies as relevant to research on aging. The others are longitudinal and historical trends. Cross- sectional trend compares performance differences between individuals from different age groups at a single time of measurement. Researchers refer to these age groups as cohorts, with cohort membership contingent on birth date in studies that sample from the population. Longitudinal trend refers to age changes within a given cohort (or cohorts) when measured at two or more times. Historical effects refer to contextual differences that relate to measurement at distinctive historical periods (cf. Rubin and Rahe, 2010, on masters swimming). Although differentiation among these trends has clear implications for quasi-experimental studies of aging in the general population, Schaie (1977) reasons that the same dimensions have comparable relevance to exploratory designs with sampling biased toward the upper or lower extremes of a normative distribution.

Confounds associated with these temporal trends include the following. Cross-sectional designs confound cohort differences and age changes. Longitudinal designs confound age changes and historical effects. Consequently, any inference that cross-sectional age differences are “virtually” the same as longitudinal age changes is true only in the absence of both cohort differences and historical change.

Schaie (2009) reports about cognitive performance that cohort differences (rather than age changes) are the main reason for discrepant findings between cross-sectional and longitudinal age trends. Because cross-sectional and longitudinal age trends in athletic performance show similar discrepancies (Young and Starkes, 2005), it is reasonable to question whether cross-sectional trends in physical performance also provide what Schaie (2009) terms “fallacious” evidence about aging effects. In attempt to provide an answer, let us consider properties of the databases.

### Archived Data on Athletic Performance

Previous research includes two main types of database relevant to athletic performance. “Ordinary” performances include those below peak levels, such as longitudinal data from samples of typical master athletes; all finishers in a given competition, etc. “All time best” performances include age class records; inclusion in Top N rankings, and top finishers in a prestigious competition. Distinctions between these types of data give rise to different expectations about age trend.

Examples of “ordinary” performance data include longitudinal archives that typify long- term master athletes without major injury during the measurement period. The data usually include personal best and less estimable performances but without a framework of interpersonal comparison to justify data inclusion. Such data are subject performance variation due to factors other than aging: for example, waxing and waning of competitive interest, general motivation, training practices, incentives to compete (cf. Medic et al., 2009) and minor injuries. The same factors may also affect performances below the top ranks of competitors in championships or regular competitions. Because aging is only one of many influences on performance in such data, it is unsurprising that performance variation with age tends to be lower than in databases restricted to best ever performance (Stones and Kozma, 1982; Young and Starkes, 2005).

Best performance archives typically collapse the age continuum into 5 years competitive categories. Moreover, the entry and retention of data depend on comparisons with age peers. For example, a record holder in a given age category may become a record holder in a later age category despite a decline in performance across times of measurement. What is important to data retention is performance time relative to age peers not the absolute level of performance. On the other hand, any entry (e.g., in a listing of age class records) may eventually undergo replacement by a subsequent superior performance by the same or another athlete. Because inclusion in such a database depends only on meeting “best ever” criteria, there is no reason to anticipate major differences between cohort differences and age changes.

A final property characteristic of database analysis relates to cohort identification. For archival data with a prolonged time period for inclusion (e.g., “all time” records or performance rankings), athletes with entries in the same age category may differ in birth date. It is for this reason that Stones and Hartin (2017) broke with tradition by defining cohort membership as age at entry into the database rather than date of birth. Differentiation of cohorts based on age at initial data entry rather than birth date seems reasonable because master athletes differ in the ages at which they begin to compete and/or achieve best ever performances (Rubin and Rahe, 2010). For example, one such classification separates master runners into lifelong athletes, those new to the sport and those returning after many years of absence (Pfitzinger and Latter, 2015).

### Analysis of Temporal Trends

Nearly all the studies of “best ever” athletic performances use general linear modeling for purposes of analysis. Such modeling assumes that the residuals are independent, which in statistical terminology implies an absence of correlated error. Correlated error is probable in data that includes repeat measures by some individuals (e.g., the same athlete holds records in multiple competitive events and/or age classes; the athlete appears more than once in annual lists of performance rankings; the athlete finishes near the top in successive championships). Furthermore, the frequency of repeat observations is substantial in archival data on “best ever” performances by master athletes. For example, approximately half the 2007 World Masters Athletics age class records in sprinting, middle distance running, race walking and jumping are multiple entries by athletes, with the other half being single entries (Stones, 2010).

Before this millennium, the use of general linear modeling is understandable because of an absence of readily accessible statistical procedures that enable appropriate analysis of repeated measures in unbalanced designs. Researchers at that time could either ignore the implications of correlated error or exclude repeated measures from the database. However, mixed linear modeling (MLM) procedures are now readily accessible that circumvent the problems of correlated error. In unbalanced designs with repeated measures, MLM procedures assume independence of residuals among individuals (i.e., athletes) rather than observations of those individuals.

### Generalizability of Age Trends

A final concern relates to the generalizability of age trends. Because the majority of studies report findings from record or top ranked performances, the generality of trends to lower levels of the ability spectrum remains a matter for conjecture. Moore (1975) considers that the age trends “comprise the marks of many individual athletes but they can be thought of as those set by a “super” runner, one who is in a top condition throughout the life span” (p. 256). He contrasted super runners with “ordinary” runners, whom he hypothesized to be slower but with a similar rate of age decline. Fair (2007) thinks that age trends based on “best ever” performances overcome methodological problems of selection bias (e.g., because of dropout) but might not generalize to those for “average” people. In contrast, Suominen (2010) reasons that selection bias (e.g., because of cohort differences) could result in overestimated age decline in peak performances compared to that in athletes of lower ability levels. However, none of these authors provide empirical support for their conjectures.

This author knows of only four studies that directly or indirectly provide data relevant to such generalizability (Seiler et al., 1998; Fairbrother, 2007; Ahmadyar et al., 2015; Nikolaidis et al., 2019). Although these studies sampled performance differently (e.g., “best ever” versus “ordinary” performances) and some have other methodological limitations, a tentative overview suggests lower performance loss with age at higher performance levels.

### The Present Study

The present study evaluates models of temporal trend and generalizability of findings in extensive array of “best ever” performances in marathon running by master athletes. Marathon races attract thousands of competitors from across the world. For example, a 2015 article by a renowned running magazine reports that approximately 1,100 marathon races in the United States attract approximately half-a-million competitors, comprising 44% women and 56% men, with respective mean ages of 36.7 years and 40.4 years (Runningusa.org, 2015). In other words, many competitors are master runners. Studies of age trend in their peak performance times in running consistently show an exponential increase with age (Moore, 1975; Stones and Kozma, 1980, 1981; Baker et al., 2003; Fair, 2007; Rittweger et al., 2009; Knechtle et al., 2014). The specific aims are to evaluate the respective effects of cohort differences, age changes, and historical influences and generalizability of cohort and age change trends across performance levels.

Table 1 summarizes the terms and their relationships in the models analyzed. The traditional model, termed here the Concurrent Age model, predicts performance (P) by age at the time of performance. In terms of date arithmetic, this expression of age equals the difference between the date of the n^th^ performance (D^n^) and date of birth (D^b^). Where P^n^ is the n^th^ performance, the model predicts this performance as follows: P^n^ = β_0_ + β_1_(D^n^ − D^b^), where β_0_ and β_1_ are constants.

**TABLE 1:** Nomenclature and expressions used in modeling.

**Nomenclature**	**Description**	**Expressions used in modeling**
Birth date	Date of birth	_D_^*b*^
Race date	Date of nth race	_D_^*n*^
Concurrent age	Age at nth race	D^n^ − D^b^
Performance	Performance in nth race	_P_^*n*^
Entry date	Date of entry into database	_D_^*e*^
Entry age	Age at entry into database	A^e^ = D^e^ − D^b^
Elapsed age	Interval between a athletes’ entry and a repeat entry	A^n^ = D^n^ − D^e^
Equation constants	Coefficients in modeling	β_0_, β_1_, β_2_, β_3_, β_4_, β_5_, β_6_, β_7_
Concurrent age model	The traditional on ^∗^10 component model	P^n^ = β_0_ + β_1_(D^n^ − D^b^)
Birth cohort model	A two-component model of birth date and race date	P^n^ = β_2_ + β_3_(D^n^) - β_4_(D^b^)
Entry age model	A two-component model of entry age and elapsed age	P^n^ = β_5_ + β_6_(A^e^) + β_7_(A^n^) = β_5_ + β_6_(D^e^ − D^b^) + β_7_(D^n^ − D^e^)

What is termed here the birth cohort model exemplifies Schaie’s (1977) perspective that date of birth identifies an important dimension in aging research. In order to retain consistency with the Concurrent Age model, the Birth Cohort model decomposes concurrent age into independent components that by date arithmetic equal its value. These components represent historical time of measurement (i.e., performance date) and birth date. They are combined in the Concurrent Age model but separated in the Birth Cohort model. Consequently, this model generates the following predictive expression: P^n^ = β_2_ + β_3_(D^n^) −β_4_(D^b^), where β_2_, β_3_, and β_4_ are constants.

Previous research by Stones and Hartin (2017) defines the cohort dimension not by birth date but by age at entry into the database. If the date of entry is D^e^, date arithmetic gives entry age (A^e^) as the difference between an athletes earliest performance date and birth date (i.e., A^e^ = D^e^ − D^b^). A further term in this Entry Cohort model refers to elapsed age (A^n^) as the interval between a repeated entry and the initial entry. Date arithmetic expresses the interval between the nth repeated entry and the entry date as follows: A^n^ = D^n^ − D^e^. This model decomposes concurrent age into components that represent a summation of entry age and elapsed age. It generates the following expressions for the nth performance: P^n^ = β_5_ + β_6_(A^e^) + β_7_(A^n^) = β_5_ + β_6_(D^e^ − D^b^) + β_7_(D^n^− D^e^), where β_5_, β_6_, and β_7_ are constants. This model thereby differentiates between entry cohort differences and age changes.

Based on prior discussion, the hypotheses for the study are as follows:

1. Performance times increase more rapidly at older age levels, with this effect greater in women than men (Stones and Kozma, 1982);
2. Models that decompose concurrent time into components (i.e., the Birth Cohort and Entry Cohort models) provide better fit to the data than the modeling without such decomposition (i.e., the Concurrent Age model);
3. The Entry Cohort model provides better fit to the data than the Birth Cohort model;
4. Performance time increases more rapidly at higher levels of the cohort and age change variables;
5. Performance times show shallower declines among faster than slower runners.

## Materials And Methods

### Data

Historically, the Veterans List of All-Time Rankings by the Association of Racing Statisticians’ (Association of Road Racing Statisticians [ARRS], 2017) provides the most comprehensive database on marathon running performances by master athletes. The data include up to 100 best ever running times per 5 years age by gender categories that meet the Association’s qualifying criteria. The performances downloaded during February 2018 date from 1963 to 2016.

The All-Time Veterans Rankings contain text files specific to 5 years age groups (40–44 to 90–94 years) for males and females. Each file includes data on performance time, the runner’s name and nationality, the date of performance, age of the runner (in years, months, days), date of birth, and place of performance. The maximal number of performances per age group by gender cell is 100, each of which meets a qualifying level intended to produce a consistent set of standards over the age ranges for each gender group. The files for males contain 100 performances for all age categories up to and including 80–84 years.

Thereafter, the number of performances is 33 for age category 85–89 years and 4 for age category 90–94 years. The files for females contain information on 100 runners for all age categories to 70–74 years. Thereafter, the numbers of entries are 69 for age category 75–79 years, 54 for 80–84 years, 27 for 85–89 years, and 6 for age category 90–94 years. Because the two 90–94 year categories contain few observations for either gender, they were collapsed into the 85–89 year category when computing divisional quartile rankings.

### Analytic Procedures

Because of a large sample size, the significance level is set at *p* < 0.01. The dependent variable in all analyses is a natural logarithmic of performance time. This logarithmic transformation builds on earlier findings that performance time in running increases exponentially with age; hence an expectation that the logarithm of performance time would closely approximate a linear relationship with age (Stones and Kozma, 1980; Baker et al., 2003). Analyses of the dependent variable use mixed linear modeling (MLM) procedures in SPSS 25. Terms common to all analyses are runners as a random intercept and days elapsed from an athlete’s initial entry as a repeated measure, with a scaled identity covariance structure for the repeated measure. Estimation of the coefficients is by maximum likelihood ratios.

For comparative purposes, separate MLM analyses of the Concurrent Age, Birth Cohort and Entry Cohort models include gender, components that identify the models (cf. Table 1) and their 2-way interactions as fixed effects. The gender term is categorical and model components are covariates centered on their grand means. Preliminary MLM analyses include models with only linear covariates to those with added quadratic covariates in order to test for any additional curvilinearity.

The main MLM analyses compare the Concurrent Age, Birth Cohort, and Entry Cohort models to ascertain which model provides best fit to the data. The estimation of goodness of fit is by deviance statistics, where deviance is the difference in –2 Log Likelihood estimates between models. Lower values for –2 Log Likelihood indicates better fit. The significance differences in -2 Log Likelihood is given by Chi squared (χ^2^) with degrees of freedom equal to differences in the number of model parameters.

A subsequent analysis adds to the best fitting model quartile rankings of performance time. These quartile rankings are within divisional groupings (i.e., age category by gender groups). The purpose of this analysis is to evaluate the generality of temporal trends across a continuum that represents the highest to lowest levels of performance. A final analysis attempts to clarify reasons that underlie the findings on generality.

## Results

### Preliminary Analyses

The data include 937 performances by 387 men performances and 856 performances by 301 women. The mean ages are 62.05 years for men (s.d. = 13.61 years) and 60.5 years for women (s.d. = 13.11), with birth dates ranging from 1895 to 1976. The mean numbers of performances per runner are 6.64 for men (s.d. = 7.13) and 6.4 for women (s.d. = 5.5). The mean Elapsed Ages are 36.71 months for men (s.d. = 55 months) and 34.34 months for women (s.d. = 46.18 months).

The distribution of the dependent variable – the natural logarithm of performance time in minutes – approximates normality. It has a mean value of 5.26 (s.d. = 0.28), with moderate skew (1.07), moderate kurtosis (0.94) and a histogram that shows only moderate departure from a normal curve. Pearson correlations of the dependent variable with Concurrent Age – in months – are 0.93 for men and 0.95 for women, which for descriptive purposes indicate strong linear age trends.

### MLM Analyses

The preliminary MLM analyses show significantly better fit for all the Concurrent Age, Birth Cohort, and Entry Cohort models after inclusion of quadratic covariates as fixed effects (all *p* < 0.01). Consequently, the following analyses report finding for models with polynomial expressions of their components. Table 2 shows goodness of fit statistics for Birth Cohort and Entry Cohort models compared to the Concurrent Age model. The findings indicate significantly better fit for the Concurrent Age than the Birth Cohort model but significantly better fit for the Entry Cohort than the Concurrent Age model.

**TABLE 2:** Goodness of fit of the birth cohort and entry cohort models compared to the concurrent age model.

**Model**	**Number of**	**Number of**	**Deviations**	**Significance of**
	**fixed**	**random**	**from -2 log**	**deviation from the**
	**effect**	**effect**	**likelihood**	**concurrent**
	**parameters**	**parameters**	**for the**	**age model**
			**concurrent**	
			**age model**	
Concurrent age	7	1	–	–
Birth cohort	12	1	51.285	<0.001
Entry cohort	12	1	–135.176	<0.001

Because of the unexpectedly low fit for the Birth Cohort model, the following analysis queries whether addition of linear and quadratic covariates relevant to Elapsed Age would improve model fit. This extended version therefore subsumes all three temporal dimensions of cohort difference, age change and historical effects. Although the findings indicate better fit for the extended over the original version of the Birth Cohort model (χ^2^ = −108.667, *df* = 2, *p* < 0.001), goodness of fit is still inferior to that for the Entry Cohort model (χ^2^ = 77.794) despite two additional degrees of freedom. For the sake of completeness, the author must mention that the addition of terms representative of historical transition (i.e., linear and quadratic performance date covariates) results in lower goodness of fit for this extended over the original Entry Cohort model despite two additional degrees of freedom (χ^2^ = 33.987). Consequently, the original Entry Cohort model provides better goodness of fit than any other model.

The coefficients in the Entry Cohort model show only minor differences after inclusion of performance level quartiles. Consequently, Table 3 includes findings for the latter. The findings indicate that performance times (1) are higher in women than men; (2) increase at a higher rate at older ages for both the entry cohort and elapsed age terms (i.e., as indicated by significant and positive quadratic coefficients; (3) increase with the interaction between the entry cohort and elapsed age terms; (4) increase more for women than men with increases in the entry cohort and elapsed age terms; (5) are expectedly higher in the slower than faster performance quartiles; and (6) increase to a greater extent in the slower than the faster quartiles with increases in entry cohort age and elapsed age.

**TABLE 3:** Fixed effect statistics for entry cohort model with performance time quartiles.

**Model term**	**Coefficient**	**significance**	**95% Confidence**	
			**interval**	
			**Lower**	**Upper**
Intercept	5.143	0.000	5.137	5.148
Female	0.182	0.000	0.175	0.188
Male	0	.	.	.
Performance quartile = 76–100	0.074	0.000	0.068	0.079
Performance quartile = 51–75	0.054	0.000	0.049	0.059
Performance quartile = 26–50	0.031	0.000	0.026	0.036
Performance quartile = 1–25	0	.	.	.
Entry cohort	−0.003	0.000	−0.003	−0.003
Entry cohort quadratic	2.91^∗^10^–6^	0.000	2.78^∗^10^–6^	3.03^∗^10^–6^
Elapsed age	0.001	0.000	0.001	0.001
Elapsed age quadratic	1.97^∗^10^–6^	0.000	1.55^∗^10^–6^	2.38^∗^10^–6^
Entry cohort ^∗^ Elapsed age	4.64^∗^10^–6^	0.000	4.33^∗^10^–6^	4.96^∗^10^–6^
Entry cohort ^∗^ FEMALE	2.33^∗^10^–4^	0.000	1.93^∗^10^–4^	2.72^∗^10^–4^
Entry cohort ^∗^ MALE	0	.	.	.
Elapsed age ^∗^ FEMALE	2.13^∗^10^–4^	0.000	1.31^∗^0^–4^	2.95^∗^10^–4^
Elapsed age ^∗^ MALE	0	.	.	.
Entry cohort ^∗^ Performance quartile = 76–100	2.77^∗^10^–4^	0.000	2.42^∗^10^–4^	3.11^∗^10^–4^
Entry cohort ^∗^ Performance quartile = 51–75	1.71^∗^10^–4^	0.000	1.38^∗^10^–4^	2.04^∗^10^–4^
Entry cohort ^∗^ Performance quartile = 26–50	7.17^∗^10^–5^	0.000	3.97^∗^10^–5^	1.04^∗^10^–4^
Entry cohort ^∗^ Performance quartile = 1–25	0	.	.	.
Elapsed age ^∗^Performance quartile = 76–100	3.81^∗^10^–4^	0.000	2.87^∗^10^–4^	4.76^∗^10^–4^
Elapsed age ^∗^ Performance quartile = 51–75	1.92^∗^10^–4^	0.000	1.02^∗^10^–4^	2.83^∗^10^–4^
Elapsed age ^∗^ Performance quartile = 26–50	2.26^∗^10^–4^	0.000	1.36^∗^10^–4^	3.16^∗^10^–4^
Elapsed age ^∗^ Performance quartile = 1–25	0	.	.	.

Final analyses by multinomial logistic regression explore reasons why slower rather than faster runners show greater performance deterioration in both cross-sectional and longitudinal trends. The data for these analyses are aggregated within athletes. The target variable is divisional performance rankings (i.e., performance time quartiles), with the fastest quartile as the reference category in an initial analysis. The fixed effect terms include entry cohort as a covariate centered on its grand mean with the elapsed age variable simplified to indicate the presence of single or multiple data entries. Alternate reference categories for the latter are multiple followed by single data entries.

**FIGURE 1:**
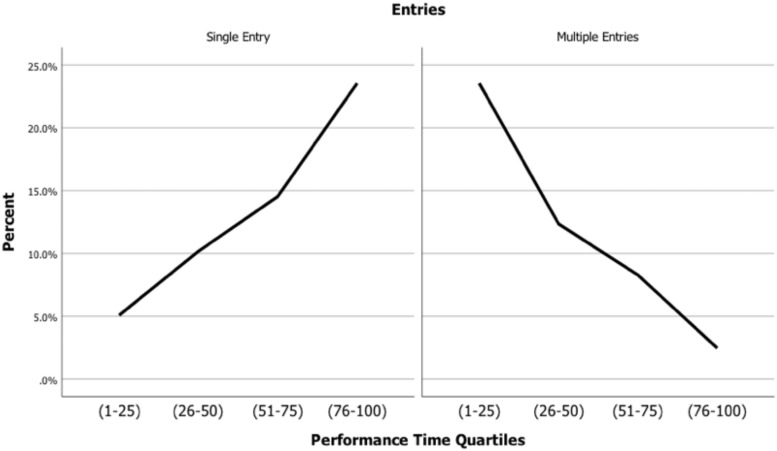
Percent single and multiple data entries by performance quartiles.

Table 4 shows findings for exponential coefficients (i.e., odds ratios). Compared to the fastest quartile, only the slowest quartile has older entry ages at *p* < 0.01. With multiple entries as the reference category for the nominal variable, the fastest quartile has the lowest likelihood of single entries. The likelihood of single entries increases significantly across quartiles, with the odds of a single entry in the slowest quartile being 42,626 times higher than in the fastest quartile. Conversely, with the slowest quartile and single data entries as reference categories, the odds of multiple entries in the fastest quartile are 42,626 times higher than in the slowest category. Figure 1 illustrates these findings.

**TABLE 4:** Regression of performance time quartiles against age at entry and single or multiple age entries.

**Performance**		**Exponential**		**95% confidence**	
**time**	**Model term**	**(coefficient)**	**Significance**	**interval for**	
**quartiles**				**Exp(coefficient)**	
				**Lower**	**Upper**
(76–100)	Intercept	0.107	0.000	0.066	0.174
	Age at entry	1.002	0.007	1.001	1.004
	Single entry	42.626	0.000	23.392	77.676
	Multiple entries	.	.	.	.
(51–75)	Intercept	0.361	0.000	0.269	0.484
	Age at entry	1.001	0.036	1.000	1.003
	Single entry	7.922	0.000	4.930	12.729
	Multiple entries	.	.	.	.
(26–50)	Intercept	0.535	0.000	0.414	0.691
	Age at entry	1.001	0.251	0.999	1.002
	Single entry	3.763	0.000	2.357	6.007
	Multiple entries	.	.	.	.

## Discussion

The best fitting model in this study includes variables termed entry cohort and elapsed age. Findings with this model show the following: (1) higher performance times in women than men; (2) higher rates of increase in performance time at older cohort ages and elapsed ages; and (3) increases in performance time with interactions between cohort age and elapsed age, cohort age and gender, and elapsed age and gender (i.e., with greater increases in women than men). Also, increases in performance time with cohort age and elapsed age are higher in slower than faster performance quartiles, with athletes in the faster quartiles more likely to have multiple data entries and athletes in the slower quartiles single data entries.

The beginning of this article includes discussion of the methodology of early studies of age trends in performances by master athletes. Such methodology falls here under a rubric of the Concurrent Age model. This approach to modeling endures today – despite historical progress in the quality of available data and more sophisticated analytic procedures. Such models have relative simplicity that satisfies scientific requirements for parsimony but may fall short on requirements for scientific precision. Criteria for the latter combine data analytic premises that are reasonable (e.g., independence of athletes rather than observations) with high goodness of fit. This study may be the first to provide quantitative comparison of the Concurrent Age model against alternative models that decompose concurrent age into components that represent different temporal trends. It is also the first to examine the generalizability of cohort differences and age changes across levels of athletic performance.

The findings replicate earlier evidence that performance decline is greater at older ages and in women than men. As hypothesized, the Entry Cohort model provides better fit to the data than the Concurrent Age and Birth Cohort models. This finding provides justification to include entry cohort and age change terms in future research. Trends on both of these components exhibit greater rates of performance decline at older ages, with the declines steeper in women than men. Finally, the findings for both cohort differences and age changes support the hypothesis of greater performance decline in slower than faster runners.

The use of performance rankings to predict performance time is compatible with earlier research (Fairbrother, 2007). That study includes analysis of U.S. Masters Swimming’s annual Top-Ten rankings for 50 m freestyle swimming. However, Fairbrother (2007) examines performance trends on concurrent age to derive inferences about generalizability from residual error. Inferences about generalizability in the present study follow from predicted scores for performance trends on entry cohort age and elapsed age. Despite these differences in methodology, the findings uniformly indicate shallower performance declines on the respective temporal dimensions by faster than slower athletes.

Moreover, analysis of the quartile rankings suggests reasons why the faster runners show shallower age declines. The fastest runners (1) are more likely to enter the database at a younger age than the slowest runners and (2) have a higher frequency of multiple data entries. An interpretation is that a combination of multiple entries by younger runners provide evidence for high levels commitment by these runners, which implies prolonged continuation of effective training practices, which in turn implies greater retention of faster racing times.

Consequently, information for coaches and master marathon runners might advisedly include planning for continued participation in order to realize and retain performance potential. Such planning could include competition against high performers in surrounding age classes, participation in races on faster courses, and extra commitment upon entry into a higher age class (Medic et al., 2009). The planning might also include adequate nutrition, sleep, and rest times in order to facilitate recovery from strenuous training and racing. Finally, coaches and master athletes might model their own practices on those of master athletes that demonstrably retain high levels of performance over prolonged historical periods.

Although the strengths of the study include analytic innovations applied to the most extensive database available on “best ever” marathon performances by master athletes, a concluding comment must also mention the study’s limitations. Analysis of any such database can only be retrospective. Master athletes may exhibit high-level or low-level performances but assignment to such conditions cannot be by random assignment. Consequently, drawing causal inferences requires caution. Moreover, the database provides information mainly on performance times and demographics. It requires more exhaustive investigations to fully understand why and how some master athletes retain elite status over prolonged historical periods.

## Data Availability Statement

The datasets generated for this study are available on request to the corresponding author.

## Ethics Statement

The study is exempt from full review by the Research Ethics Board of Lakehead University because all the data analyzed are in the public domain.

## Author Contributions

MS analyzed the data and wrote the manuscript.

## Conflict of Interest

The authors declare that the research was conducted in the absence of any commercial or financial relationships that could be construed as a potential conflict of interest.
